# Factors influencing sialorrhea in orally intubated patients: a lasso and logistic regression analysis

**DOI:** 10.3389/fmed.2025.1623226

**Published:** 2025-08-18

**Authors:** Jinlei Du, Min Wang, Xiaoling Wu, Tianbo Yu, Jiquan Zhang, Jimin Wu

**Affiliations:** ^1^Department of Critical Care Medicine, Zigong Fourth People's Hospital, Zigong, China; ^2^Department of Critical Care Medicine, Deyang People's Hospital, Deyang, China; ^3^Department of Critical Care Medicine, Xiangyang Central Hospital, Xiangyang, China

**Keywords:** endotracheal intubation, sialorrhea, prevalence, risk factors, logistic regression analysis

## Abstract

**Objective:**

To investigate the prevalence of sialorrhea in orally intubated patients and systematically analyze its influencing factors.

**Methods:**

A cross-sectional study was conducted from March 15 to 31, 2025, involving 40 tertiary general hospitals across 10 prefecture-level cities in Sichuan Province, including Chengdu, Zigong, and Mianyang. The investigation assessed the current status of sialorrhea in patients undergoing oral endotracheal intubation.

**Results:**

A total of 453 questionnaires were collected, of which 440 were valid, yielding an effective response rate of 97.0%. Statistical analysis revealed that the incidence rate of sialorrhea among orally intubated patients was 27.27%. Multivariate logistic regression analysis identified the following as independent risk factors for sialorrhea: Body Mass Index (BMI) (OR = 1.365, 95% CI: 1.217–1.531), Smoking (OR = 8.944, 95% CI: 4.272–18.727), Number of Combined Functional Impairment Systems (OR = 2.844, 95% CI: 1.814–4.460), Combined Oral Disease (OR = 2.578, 95% CI: 1.240–5.359), and Neurological Diseases (OR = 4.040, 95% CI: 1.053–15.507). A restricted cubic spline analysis further demonstrated that when BMI exceeds 22.785, the risk of developing sialorrhea increases significantly.

**Conclusion:**

The incidence of sialorrhea in orally intubated patients is at a moderate-to-low level. This condition is closely associated with elevated BMI, smoking, a higher number of combined functional impairments, the presence of oral disease, and underlying nervous system disorders.

## 1 Introduction

In recent years, orotracheal intubation has become one of the most effective interventions for critically ill patients, with a clinical utilization rate exceeding 90% (1). However, the widespread use of this technique has been accompanied by an increasing incidence of complications. Among these, sialorrhea is a relatively common but often overlooked complication in patients undergoing orotracheal intubation (2). Sialorrhea, also referred to as drooling, is a syndrome characterized by the overflow or pooling of saliva in the oral cavity due to oropharyngeal dyskinesia, sensory coordination disorders, and impaired swallowing reflexes (3). Studies have reported that sialorrhea can result in a range of adverse outcomes in intubated patients, including perioral skin and/or mucosal infections, halitosis, and oral microbiota imbalance (4). Moreover, it significantly increases the risk of aspiration, unplanned extubation, and ventilator-associated pneumonia (VAP), thus prolonging mechanical ventilation duration and escalating healthcare costs (5).

Currently, most clinical research on sialorrhea has focused on children with cerebral palsy and patients with stroke, primarily addressing its risk factors, intervention strategies, and treatment outcomes (6, 7). However, there are few studies that specifically investigate the prevalence and influencing factors of sialorrhea in patients undergoing orotracheal intubation. Given the significant impact of sialorrhea on patient outcomes and the increasing utilization of orotracheal intubation in critical care settings, there is a pressing need to address this gap in the literature. Understanding the prevalence and risk factors of sialorrhea in orally intubated patients is essential for developing targeted interventions and reducing associated complications. Therefore, this study aims to conduct a cross-sectional survey to assess the current status of sialorrhea in this population and perform a comprehensive analysis of its associated risk factors. By generating clinically relevant evidence, we aim to support early risk identification, guide preventive strategies, and ultimately improve the quality of airway management and patient outcomes in the ICU setting.

## 2 Subjects and methods

### 2.1 Study participants

This cross-sectional study was conducted from March 15 to March 31, 2025. A convenience sampling method was used to select patients undergoing orotracheal intubation in intensive care units (ICUs) of 40 tertiary general hospitals located in 10 prefecture-level cities in Sichuan Province, including Chengdu, Zigong, Mianyang, Deyang, Suining, Neijiang, Yibin, Leshan, Nanchong, and Guangyuan.

Inclusion criteria: (1) patients aged ≥18 years; (2) patients or their legal representatives voluntarily agreed to participate in this study. Exclusion criteria: (1) patients with a history of pharyngeal or laryngeal surgery; (2) patients with hyperthyroidism; (3) patients with autoimmune disorders; (4) patients with hemodynamic instability; (5) pregnant women or those in the perinatal period.

However, there are few studies that specifically investigate the prevalence and influencing factors of sialorrhea in patients undergoing orotracheal intubation. The results indicated that approximately 30% of the patients exhibited signs of sialorrhea. Based on the sample size estimation formula for cross-sectional studies, N=μ2α2⋅P⋅(1−P)E2 , where N is the required sample size, Zα2 represents the Z-score for a 95% confidence interval (1.96), P is the expected prevalence (30%), and E is the allowable margin of error (0.05), the required sample size was calculated to be approximately 322. Considering a potential 10% rate of invalid responses, the final sample size was determined to be 354 participants.

### 2.2 Ethical consideration

This study was conducted in accordance with the ethical standards outlined in the National Statement on Ethical Conduct in Human Research (2016) and the principles of the Declaration of Helsinki. Ethical approval for this study was obtained from the Ethics Committee of Zigong Fourth People’s Hospital, approval number EC-2025-104.

Given that all participants were undergoing orotracheal intubation, and a proportion of them were in a comatose or otherwise incapacitated state, informed consent was obtained either directly from the patient or, when the patient was unable to provide consent, from their legal guardian or family member. Participation was entirely voluntary, and participants or their legal representatives were informed that they could withdraw from the study at any point without any consequences. All surveys and assessments were conducted in a safe environment to ensure participant comfort and data security. All data collected during the study were anonymized to protect participant privacy.

### 2.3 Methods

#### 2.3.1 Diagnostic criteria for sialorrhea

Currently, there is no internationally established diagnostic standard specifically for sialorrhea in patients undergoing orotracheal intubation. Therefore, this study adopted the widely accepted metric of Unstimulated Salivary Flow Rate (USFR) for evaluation. According to Miranda-Rius et al. (8), the normal USFR for healthy adults ranges between 0.3 and 0.5 mL/min. A rate below 0.1 mL/min is indicative of salivary gland hypofunction, whereas a consistently elevated rate beyond the upper limit of normal suggests excessive salivary secretion.

In this study, a USFR > 0.5 mL/min was used as the objective diagnostic threshold for sialorrhea, defined as a salivary secretion rate significantly exceeding normal physiological levels in a resting state. All patients were assessed without any prior oral stimulation to ensure that the measurements reflected natural resting conditions. Saliva was collected using a standardized procedure: at predetermined time intervals, a 20 mL syringe was connected to a suction catheter and inserted through the central hole of a bite block or the lateral port of the endotracheal tube into the oral and pharyngeal cavity. Manual suction was used to collect the saliva. The USFR was calculated using the following formula: USFR = Volume of Saliva Collected (mL)/Time (min). Based on this criterion, patients were categorized into a sialorrhea group (USFR > 0.5 mL/min) and a non-sialorrhea group (USFR ≤ 0.5 mL/min) for comparative analysis.

The choice of manual saliva collection was primarily driven by its feasibility and cost-effectiveness in a clinical setting. This method is relatively simple to perform and does not require specialized equipment, making it suitable for use in a variety of healthcare environments, including those with limited resources. Moreover, manual collection enables immediate measurement of saliva volume, which is important in clinical contexts requiring timely evaluation and decision-making. To ensure the accuracy and consistency of saliva collection, all research staff involved in this procedure received standardized training prior to the study. Only those with at least 3 years of experience in intensive care medicine were selected to perform the saliva collection, as their expertise helps minimize potential variability in the manual suction process. Despite these measures, we acknowledge that manual saliva collection may introduce some degree of measurement error. Future studies may benefit from incorporating more automated or technology-assisted methods to enhance the precision and objectivity of saliva flow assessment.

#### 2.3.2 Data collection

A general information questionnaire was developed by the research team based on literature review and clinical experience to collect demographic and disease-related data. Demographic data included sex, age, educational level, body mass index (BMI), and marital status. In this study, married status referred to individuals currently in a marital relationship, while unmarried status encompassed those who were single, divorced, or widowed.

Disease-related data included primary diagnosis, Acute Physiology and Chronic Health Evaluation II (APACHE II) score, presence of oral diseases, number of concomitant organ system dysfunctions, and Glasgow Coma Scale (GCS) score. Oral diseases in this study referred to dental pulp diseases, oral mucosal diseases, oral surgical conditions, and periodontal diseases (9). The number of organ system dysfunctions referred to comorbid conditions outside of the primary diagnosis, such as cerebral infarction or hemorrhage (nervous system), respiratory failure or chronic obstructive pulmonary disease (respiratory system), and shock or hypertension (circulatory system).

As all patients in this study were undergoing orotracheal intubation and were therefore unable to speak normally, the GCS scores were calculated based only on eye-opening and motor responses, with the verbal response uniformly recorded as “T” (indicating endotracheal intubation). The depth of intubation was recorded as the distance from the patient’s incisors. All data were obtained either from the patient’s medical records or by interviewing family members.

### 2.4 Survey methods and quality control

Prior to the commencement of the project, a dedicated research team was established, and one liaison officer was appointed at each participating institution. All liaison officers received standardized training from the team members. The training covered the research background, objectives, survey methodology, and the criteria for assessing drooling symptoms. After the training, the project leader distributed the survey questionnaires to each medical institution through courier services or direct delivery. Within each institution, the liaison officer was responsible for overseeing the quality control of the survey process, including the distribution and collection of questionnaires and conducting random assessments of completed surveys.

In this study, all research staff involved in data collection had at least 3 years of experience in intensive care medicine. After all questionnaires were collected, the project leader performed random sampling for quality monitoring, and questionnaires with potential risk biases were excluded. Additionally, two students specializing in medical statistics independently verified and entered all survey data. To ensure the accuracy of the study results, all statistical analyses were reviewed and guided by experts in statistical medicine from our hospital.

### 2.5 Statistical methods

Data were entered by two researchers using a double-checking method, initially organized using EpiData 3.0, and then analyzed using SPSS 26.0 and R software. Categorical data were described using frequency and percentage. Continuous data were presented as mean ± standard deviation (SD) if normally distributed, and as median and interquartile range (IQR) if not normally distributed.

To improve the robustness of variable selection, LASSO (Least Absolute Shrinkage and Selection Operator) regression analysis was used for initial screening of characteristic variables. This method applies L1 regularization penalties to the regression coefficients, allowing for variable selection and model shrinkage. It effectively prevents multicollinearity and enhances the model’s generalizability. Variables with non-zero coefficients selected from LASSO were further included in binary logistic regression analysis to assess their independent effects on drooling symptoms in patients undergoing orotracheal intubation.

Before logistic regression modeling, all candidate variables underwent multicollinearity diagnostics. Variance inflation factors (VIF) were used to assess correlations between variables. If VIF > 10, severe multicollinearity was assumed, and the variable was excluded. The goodness of fit of the model was assessed using the Hosmer-Lemeshow test; *p* > 0.05 indicated good model fit. Additionally, to explore the non-linear relationship between continuous variables and the risk of drooling, continuous variables that showed statistical significance in the logistic regression model were selected for restricted cubic spline (RCS) modeling, followed by visual analysis. This method helps reveal the complex associations between variables and outcomes, providing more accurate clinical risk identification. All tests were two-sided, and a *p*-value < 0.05 was considered statistically significant.

## 3 Results

### 3.1 General patient information

A total of 460 questionnaires were distributed, and 453 were returned. Among the 453 returned questionnaires, 6 were excluded due to unclear handwriting, 4 due to repeated corrections, and 3 due to incomplete information. After discussion by the project team, these 13 questionnaires were discarded. As a result, 440 valid questionnaires were collected, yielding an effective response rate of 97%. To ensure the reliability of the study conclusions and the precision of overall parameters, the project team decided to include all 440 valid questionnaires in the statistical analysis.

**Figure 1:**
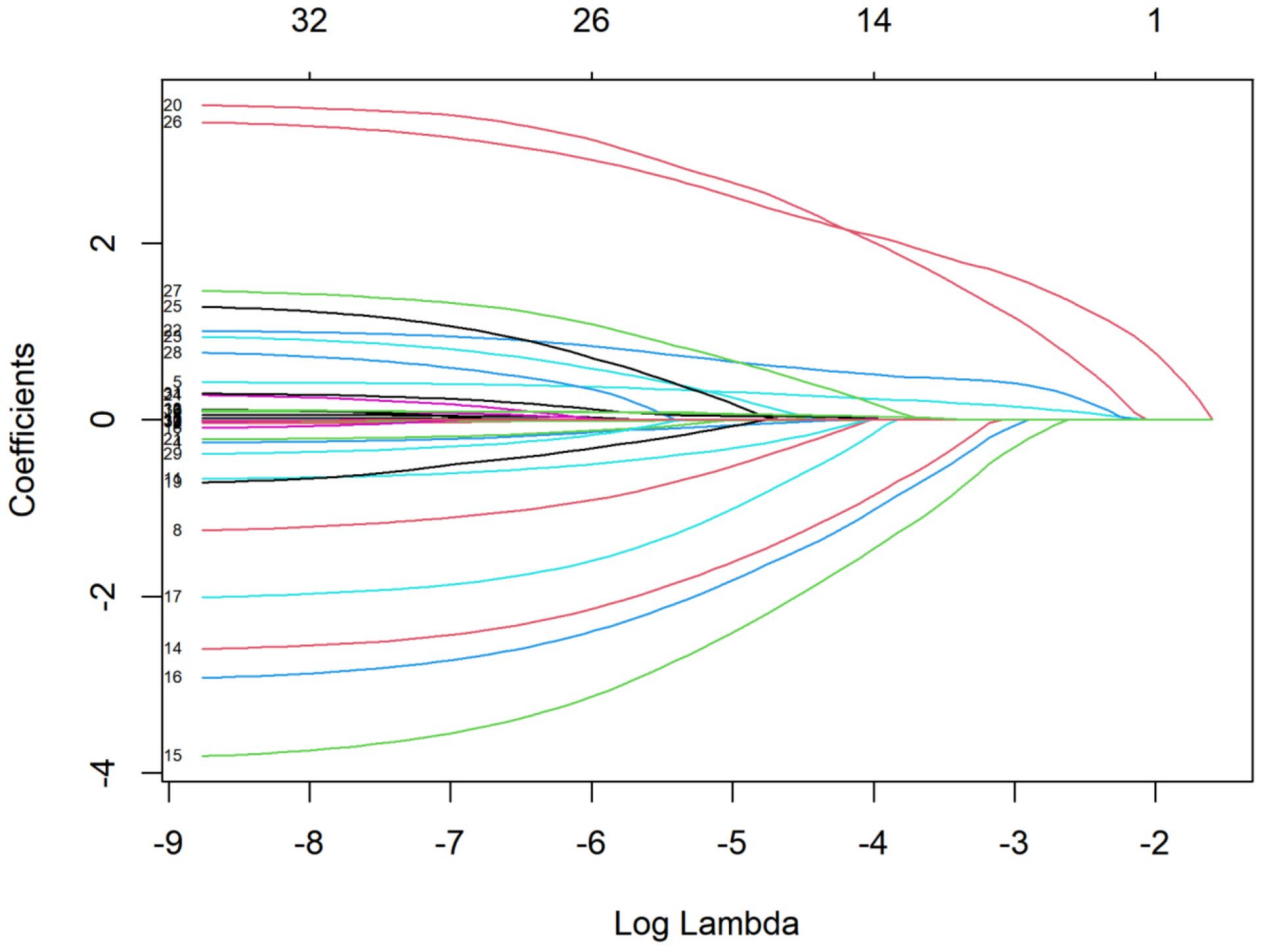
LASSO coefficient path for variables in assessing sialorrhea in orally intubated patients for details.

Among the 440 patients, 216 (49.09%) were male and 224 (50.91%) were female. The average age of the patients was 59 years. Of these, 120 patients developed sialorrhea, and the unstimulated salivary flow rate was 0.71 mL/min. The incidence of sialorrhea was 27.27%. LASSO regression analysis revealed that age, education level, smoking, BMI, primary disease, number of combined functional impairment systems, combined oral disease, intubation depth, catheter diameter, and number of ICU admissions were included in the regression model. Potential influencing variables for sialorrhea in orally intubated patients were selected using LASSO regression (Table 1 and Figure 1).

**Table 1:** General characteristics of patients and variables selected by LASSO analysis for assessing sialorrhea in orally intubated patients.

Variable	Group	Sample size with sialorrhea (*n* = 120)	Sample size without sialorrhea (*n* = 320)	LASSO coefficient	Included in model
Gender no. (%)	Male	66(55.00)	150(46.88)	0.000	No
	Female	54(45.00)	170(53.12)		
Age (years, mean ± SD)		57.23 ± 16.25	59.70 ± 18.56	0.008	Yes
Education level [*n* (%)]	Primary school or below	38(31.67)	111(34.67)	0.302	Yes
	Junior high school	39(32.50)	95(29.69)		
	Senior high school	26(21.67)	62(19.38)		
	College or above	17(14.16)	52(16.26)		
				
Marital status [*n* (%)]	Married	94(78.33)	251(78.44)	0.022	Yes
	Unmarried	26(21.67)	69(21.56)		
Smoking [*n* (%)]	No	55(45.83)	284(88.75)	2.590	Yes
	Yes	65(54.17)	36(11.25)		
BMI (kg/m^2^, mean ± SD)		24.66 ± 3.77	22.37 ± 3.16	0.322	Yes
GCS score (mean ± SD)		6.92 ± 1.86	6.44 ± 1.67	0.000	No
Primary disease [*n* (%)]	Respiratory system disease	20(16.67)	86(26.88)	2.520	Yes
	Neurological Diseases	74(61.67)	39(12.19)		
	Circulatory system disease	12(10.0)	92(28.75)		
	Digestive system disease	10(8.33)	86(26.88)		
	Other system disease	4(3.33)	17(5.30)		
APACHE II score (mean ± SD)		20.57 ± 2.38	19.99 ± 2.47	0.075	Yes
Number of combined functional impairment systems [*n* (%)]	1 system	5(4.17)	40(12.50)	2.750	Yes
	2 systems	33(27.50)	127(39.69)		
	3 systems	39(32.50)	114(35.63)		
	≥4 systems	43(35.83)	39(12.18)		
Combined oral disease [*n* (%)]	No	61(50.83)	256(80.00)	0.681	Yes
	Yes	59(49.17)	64(20.00)		
Intubation days (days, mean ± SD)		6.05 ± 4.54	5.85 ± 5.03	0.000	No
Intubation depth (cm, mean ± SD)		23.93 ± 1.24	23.48 ± 1.61	0.000	No
Intubation process [*n* (%)]	1-time success	66(55.00)	271(84.69)	0.044	Yes
	2-time success	26(21.67)	28(8.75)		
	≥3-time success	28(23.33)	21(6.56)		
Catheter diameter [*n* (%)]	6.0 mm	7(5.83)	14(4.38)	0.588	Yes
	6.5 mm	4(3.33)	24(7.50)		
	7.0 mm	42(35.00)	99(30.93)		
	7.5 mm	50(41.67)	136(42.50)		
	8.0 mm	17(14.17)	47(14.69)		
Catheter type [*n* (%)]	Conventional catheter	72(60.00)	177(55.31)	0.000	No
	Reinforced catheter	30(25.00)	81(25.31)		
	Flushable catheter	18(15.00)	62(19.38)		
Number of ICU admissions [*n* (%)]	1 time	85(70.83)	235(73.44)	0.727	Yes
	2 times	33(27.50)	67(20.94)		
	≥3 times	2(1.67)	18(5.62)		
Sedation and/or analgesia days (days, mean ± SD)		7.50 ± 4.55	6.60 ± 5.22	0.068	Yes
Enteral nutrition treatment [n (%)]	No	23(19.17)	103(32.19)	0.000	No
	Yes	97(80.83)	217(67.81)		

### 3.2 Multivariable analysis of sialorrhea in orally intubated patients

Using the assessment results of sialorrhea in orally intubated patients as the dependent variable, the independent variables selected through LASSO analysis were included in the regression model for multivariable logistic regression analysis. Prior to the analysis, collinearity diagnostics were conducted on the variables that showed statistical differences in the univariate analysis. The tolerance of each model was found to be greater than 0.1, and the variance inflation factor (VIF) was less than 10, indicating no multicollinearity between the variables (10).

**Figure 2:**
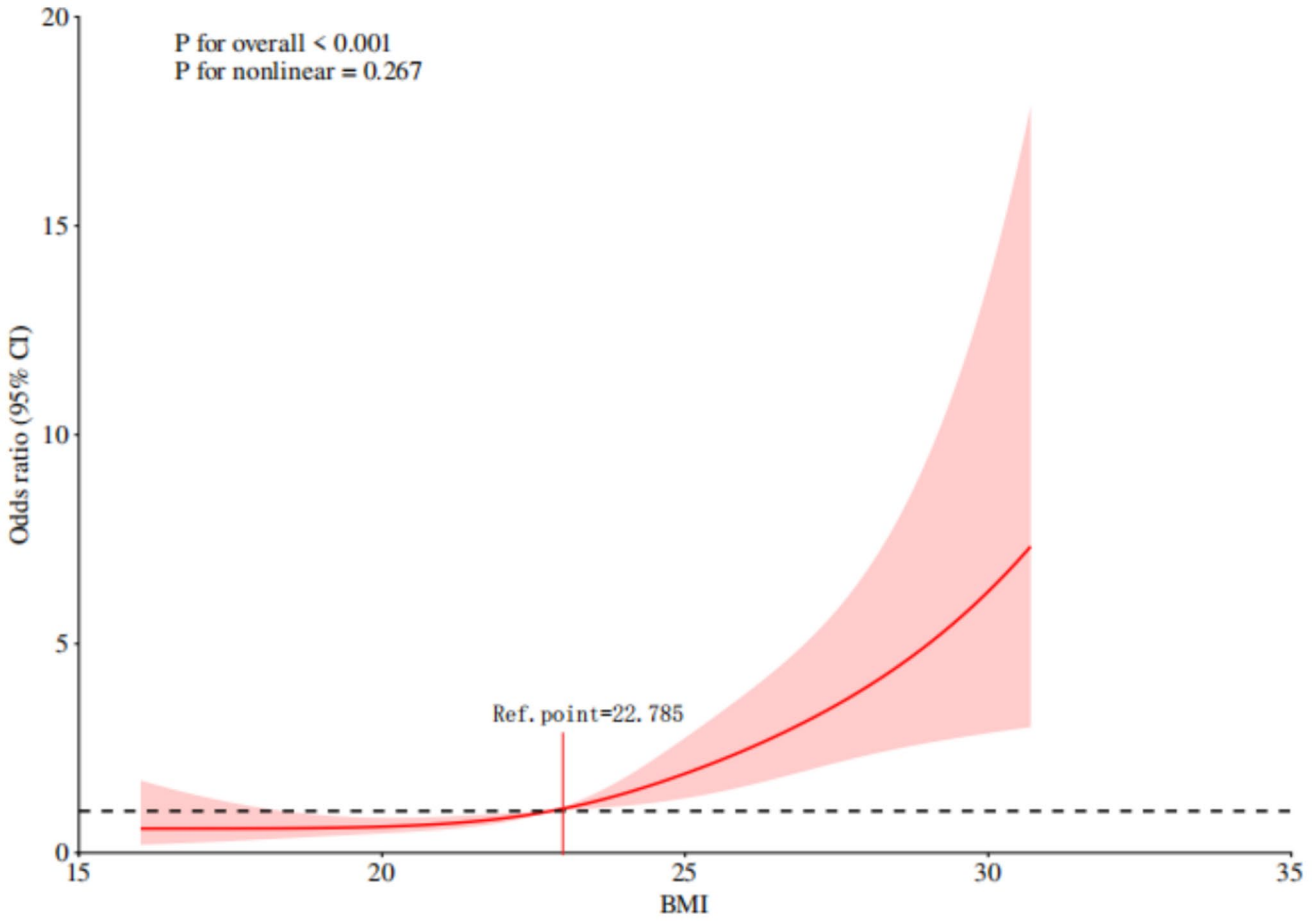
Restricted cubic spline analysis of the association between BMI and sialorrhea in orally intubated patients.

The results of the multivariable logistic regression analysis showed that the following factors were independent risk factors for the occurrence of sialorrhea in orally intubated patients: BMI (OR = 1.365, 95% CI = 1.217–1.531)、Smoking (OR = 8.944, 95% CI = 4.272–18.727), Number of Combined Functional Impairment Systems (OR = 2.844, 95% CI = 1.814–4.460), Combined Oral Disease (OR = 2.578, 95% CI = 1.240–5.359), Neurological Diseases (OR = 4.040, 95% CI = 1.053–15.507). The results of the multifactorial analysis are detailed in Table 2. Further visual analysis using a restricted cubic spline (RCS) model for continuous variables revealed that when BMI exceeds 22.785, the risk of sialorrhea significantly increases in orally intubated patients. This relationship is shown in Figure 2.

**Table 2:** Multifactorial analysis of influencing factors for sialorrhea in orally intubated patients.

Variable	Category	B	SE B	P	OR	95%CI
BMI		0.311	0.059	<0.001	1.365	1.217–1.531
Smoking		2.191	0.377	<0.001	8.944	4.272–18.727
Number of combined functional impairment systems		1.045	0.230	<0.001	2.844	1.814–4.460
Combined oral disease		0.947	0.373	0.011	2.578	1.240–5.359
Primary disease	Respiratory system disease*					
	Neurological diseases	1.396	0.686	0.042	4.040	1.053–15.507

*Served as the reference group, Hosmer-Lemeshow Goodness-of-fit Test: χ^2^ = 11.220, *p* = 0.190.

## 4 Discussion

### 4.1 Prevalence and multivariable analysis of sialorrhea in orally intubated patients

This study, conducted across 40 tertiary general hospitals in 10 prefecture-level cities in Sichuan Province, investigated 440 patients who underwent oral tracheal intubation in intensive care units. The results indicated that 27.27% of the patients experienced sialorrhea. Compared with patients with amyotrophic lateral sclerosis (ALS), who have a reported prevalence of sialorrhea around 30.8% (11). The incidence rate in our study population is relatively lower. This difference may be attributed to the distinct underlying pathophysiological mechanisms between these two groups. ALS patients often suffer from progressive neurodegenerative changes that directly affect the neural control of salivary secretion, whereas our study population, comprising a broader range of critically ill patients, may exhibit more variable responses to orotracheal intubation and associated complications. Although the prevalence rate of this condition may seem relatively low, its consequences, including oral microbial imbalance, unplanned extubation, and aspiration, should not be underestimated (12). These complications can significantly impact patient outcomes and prolong recovery.

Therefore, it is crucial to implement a comprehensive management strategy for sialorrhea in orally intubated patients to mitigate the risk of adverse outcomes. Preventive measures, regular assessments, and timely interventions are necessary to reduce the occurrence of these adverse events. Such strategies would not only improve the comfort and quality of life for intubated patients but also enhance the effectiveness of mechanical ventilation therapies. Further research into optimizing these approaches will be essential in improving the overall management of mechanically ventilated patients.

### 4.2 Influencing factors of sialorrhea in orally intubated patients

#### 4.2.1 Higher BMI increases the risk of sialorrhea

The results of this study indicate that BMI (OR = 1.365) is a significant risk factor for the development of sialorrhea in orally intubated patients, suggesting that body weight plays a key role in the pathogenesis of this complication. Obesity not only affects systemic metabolic conditions but also alters the local oral microenvironment (13). Existing studies have shown that individuals with obesity have a higher oral microbial load compared to those with normal weight (14). This includes a greater quantity of microorganisms, as well as a more diverse range of species, particularly in terms of anaerobic bacteria and inflammation-associated strains. This imbalance in the oral microbiota may exacerbate inflammation, stimulate salivary gland secretion, or impair the mucosal barrier, thereby inducing or worsening sialorrhea (15). However, it is important to note that while our findings suggest an association between elevated BMI and increased risk of sialorrhea, the relationship between obesity-related oral microbiota changes and sialorrhea has not been causally proven. Further research is needed to explore the underlying mechanisms and establish a causal link.

To better understand the impact of BMI on sialorrhea risk, this study further utilized a restricted cubic spline model for continuous variable analysis. The results revealed that when BMI exceeds 22.785, the risk of sialorrhea significantly increases, showing a clear dose-effect relationship. This finding suggests that during the management of tracheal intubation and mechanical ventilation, clinical teams should pay special attention to patients’ nutritional status and physical condition. For patients with a higher BMI, enhanced oral hygiene and nursing interventions should be prioritized. Increasing the frequency of oral care can help prevent abnormal microbial growth in the mouth and the subsequent increase in secretions, thus reducing the incidence of sialorrhea and improving overall patient comfort and prognosis.

#### 4.2.2 Smoking increases the risk of sialorrhea

The study results show that smoking (OR = 8.944) is a significant risk factor for sialorrhea in orally intubated patients, indicating its considerable impact on airway management-related complications. Long-term smoking can cause persistent damage to the oral environment through various mechanisms. Nicotine, carbon monoxide, and other harmful tobacco components can significantly alter the composition of the oral microbiota, increasing the proportion of pathogenic bacteria and triggering an imbalance in the oral flora (16). Additionally, these substances can promote oxidative stress in the oral mucosal epithelial cells, leading to a chronic inflammatory state that induces conditions such as gingivitis, periodontitis, and mucosal micro-injuries. This stimulates abnormal salivary gland activity, resulting in increased salivation (17).

It is worth noting that during tracheal intubation and mechanical ventilation, patients’ ability to self-clean the oral cavity is compromised, making it difficult to eliminate oral inflammation and secretions, which further exacerbates sialorrhea symptoms (18). This study suggests that for patients with a history of smoking who undergo tracheal intubation, clinical teams should strengthen oral management strategies. This includes regular professional oral cleaning, and depending on the patient’s mucosal condition, appropriately using anti-inflammatory medications and mild mouthwashes (e.g., sodium bicarbonate, hydrogen peroxide solutions) to adjust the oral pH, inhibit bacterial growth, and maintain oral microenvironment stability (19). Such measures can effectively reduce the risk of sialorrhea.

#### 4.2.3 The more combined functional impairment systems, the higher the risk of sialorrhea

The study results indicate that the number of combined functional impairment systems (OR = 2.844) is closely associated with the occurrence of sialorrhea in orally intubated patients, making it one of the important risk factors for this complication. An increase in the number of functional impairments often suggests a higher level of complexity in the patient’s overall condition, involving the decline or failure of multiple organ functions (20). Clinically, this requires a comprehensive assessment and the use of multiple drugs for intervention.

Many drugs commonly used in clinical support treatments, such as expectorants for the respiratory system (e.g., ambroxol, acetylcysteine), sedative-hypnotics for the central nervous system (e.g., olanzapine, clonazepam), and neurotrophic agents for post-stroke conditions (e.g., citicoline), may contribute to increased salivation or disruption of salivary gland regulation through their effects on neural control mechanisms or direct stimulation of the salivary glands (21). Furthermore, as the number of functional impairment systems increases, the probability of polypharmacy and cumulative drug doses rises, and drug interactions may exacerbate oral mucosal irritation and glandular stress responses, thereby increasing the risk of sialorrhea (22).

However, it is important to note that our study did not collect data on medication use, and thus these interpretations are speculative and based on existing literature. Future studies should include detailed data on medication use to better understand the impact of drugs on sialorrhea in orally intubated patients. Given these limitations, it is particularly important for clinical practice to be cautious in the management of patients with multiple functional impairments.

Therefore, for intubated patients with multiple combined functional impairments, it is recommended that treatment plans carefully weigh the therapeutic effects and potential side effects of medications, aiming to minimize the use of drugs that may trigger abnormal glandular secretion. Additionally, enhancing the assessment and management of oral secretions in such patients can help prevent and alleviate sialorrhea-related symptoms, improving comfort and safety during mechanical ventilation.

#### 4.2.4 The risk of sialorrhea is higher in patients with oral diseases

The study results show that the presence of combined oral diseases (OR = 2.578) is one of the significant risk factors for the occurrence of sialorrhea in orally intubated patients. This conclusion is consistent with the findings of Proctor (23), which highlighted the relationship between oral health and salivary gland function. The main mechanism behind this is that when there is chronic or acute inflammation in the oral cavity, inflammatory stimuli activate nerve endings and their associated axonal reflexes, inducing epithelial cell contraction and enhancing the secretory activity of the glands, leading to a significant increase in salivation. Additionally, damage to the barrier function of the oral mucosa further exacerbates the gland’s response to inflammatory factors, causing an imbalance in the salivary regulation mechanism, which in turn triggers or worsens sialorrhea symptoms (24).

For orally intubated patients undergoing mechanical ventilation, airway patency and oral hygiene management are closely linked. If oral inflammation is not effectively controlled, it may not only increase salivation but also raise the risk of aspiration and respiratory infections (25). Therefore, for patients with combined oral diseases, it is recommended to strengthen oral assessments and care during mechanical ventilation, carefully select appropriate antimicrobial, anti-inflammatory medications, or treatments that promote mucosal repair, and actively improve the local oral environment. This approach can help reduce the inflammatory impact on the salivary glands and prevent the occurrence and progression of sialorrhea.

#### 4.2.5 The risk of sialorrhea is higher in patients with neurological diseases

The results of this study suggest that neurological diseases (OR = 4.040) are a significant risk factor for the occurrence of sialorrhea in orally intubated patients. These patients often experience disturbances in the neuro-muscular regulatory mechanisms due to damage or dysfunction of the nervous system, which interferes with the autonomic control of salivary gland secretion, leading to reflexive increases in salivation (26). Additionally, these patients often require the use of medications for cerebrovascular accidents, sedatives, and muscle relaxants during mechanical ventilation, and these medications may further exacerbate the imbalance in salivary secretion by acting on the central nervous system or peripheral nerve pathways, thereby increasing the risk of sialorrhea.

The findings of this study align with those of Naeem et al. (27), who also noted that neurological dysfunction significantly increases the incidence of sialorrhea. Based on these findings, it is recommended that healthcare providers closely monitor the oral secretion status of patients with neurological diseases undergoing orotracheal intubation. Dynamic assessment of sialorrhea risk should be conducted based on changes in salivation, and timely interventions should be implemented as necessary to enhance treatment safety and reduce the incidence of complications.

## 5 Conclusion

In conclusion, the results of this study indicate that the incidence of sialorrhea in orally intubated patients is generally low to moderate, but its occurrence is closely related to various individual factors, particularly an elevated BMI, primary neurological diseases, multiple system functional impairments, oral diseases, and a history of smoking. These factors may induce sialorrhea through mechanisms such as altering the regulation of salivary gland secretion, disrupting the oral microbiome, or enhancing local inflammatory responses.

Therefore, clinical practice should focus on strengthening oral hygiene and nursing interventions for patients with these high-risk factors, and optimize nursing processes to reduce the risk of sialorrhea. At the same time, research on interventions for sialorrhea in intubated patients remains relatively limited. Future studies should further explore the application and feasibility of non-pharmacological therapies, including cognitive behavioral therapy, low-frequency electrical stimulation, muscle vibration, acupuncture, and oral massage interventions, to construct individualized, diversified nursing strategies. This would provide evidence-based support to enhance patient comfort and treatment adherence.

## 6 Limitations

This study utilized a convenience sampling method for sample selection, which, while highly feasible in practice, may introduce sampling bias and limit the generalizability of the findings. Future research should consider conducting large-scale validation studies using multi-center, random sampling approaches to improve the external validity of the results. Additionally, due to the complex and rapidly progressing conditions of critically ill patients, this study focused on variables based on the systems involved in primary diseases during the multivariate analysis. However, it did not explore the differential impact of various diseases within the same system or the interactions between diseases across different systems on the occurrence of sialorrhea. Therefore, further research is needed to refine and validate the specific mechanisms and extent of how particular diseases affect sialorrhea in orally intubated patients.

## Data Availability

The raw data supporting the conclusions of this article will be made available by the authors, without undue reservation.
